# Requirement of transcription factor NME2 for the maintenance of the stemness of gastric cancer stem-like cells

**DOI:** 10.1038/s41419-021-04234-1

**Published:** 2021-10-09

**Authors:** Yaxin Qi, Jun Wei, Xiaobo Zhang

**Affiliations:** 1 College of Life Sciences and Laboratory for Marine Biology and Biotechnology of Pilot National Laboratory for Marine Science and Technology (Qingdao), Zhejiang University, Hangzhou, 310058 People’s Republic of China; 2 Chengdu No.7 Ba Yi School, Chengdu, 610036 People’s Republic of China

**Keywords:** Cancer stem cells, Cancer stem cells

## Abstract

Cancer stem cells (CSCs), which can self-renew and produce heterogeneous cancer cells, are the key factors during tumorigenesis. Transcription factors take essential effects on CSCs. However, the role of transcription factors in regulating the stemness of gastric cancer stem-like cells has not been well explored. In this investigation, it was found that transcription factor NME2 (NME/NM23 nucleoside diphosphate kinase 2) was upregulated in gastric cancer stem-like cells that sorted from the solid tumors of patients with gastric cancer and gastric cancer cell lines. NME2 could preserve the stemness of gastric cancer stem-like cells via suppressing their apoptosis. In vitro and in vivo data revealed that NME2 was crucial for maintaining the stemness of gastric cancer stem cells by enhancing the expression of anti-apoptosis genes. Consequently, our data contributed a new perspective to the relationship between transcription factor and the stemness maintenance of gastric cancer stem cells.

## Introduction

Nowadays, gastric cancer has become the fifth highest diagnosed cancer and the third major cause of death for adults in the world [1]. The interactions between environmental and host-associated factors are the main causes of high gastric cancer mortality, including the late clinical manifestations, underlying biological mechanism, and genetic heterogeneity [2]. Despite noticeable improvement in traditional treatments for gastric cancer, it is still mortal for many patients because of cancer progression, metastasis, and recurrence [3]. Due to the limitations of traditional treatment, researchers have begun to pay attention to the genetic and molecular mechanisms of tumor initiation and drug resistance [3]. More and more evidences show that cancer stem cells (CSCs) are located at the top of the hierarchical organizational structure of tumors. Therefore, successful eradication of CSCs may be the most promising treatment for cancer [3]. Therefore, understanding CSCs may shed light on revealing the mechanisms of cancer initiation and progression, as well as the development of novel cancer therapies [4].

At present, gene mutation, cell fusion, and cell microenvironment are considered to be the main origins of CSCs [4–8]. In recent years, a great number of studies have shown that transcription factors are abnormally expressed in CSCs [9]. The abnormal activation of transcription factors can promote the stemness and inhibit the differentiation of CSCs, suggesting that the abnormal expression of transcription factors is associated with the origin of CSCs. Transcription factors can bind to short specific DNA sequences, which are usually in the enhancers or promoters of its target genes [10], and consequently offer significant contributions to tumorigenesis [11]. In CSCs, numerous transcription factors, such as Nanog, OCT4, and SOX2, are overexpressed, which is in common with early embryonic stem cells (ESCs) [11]. It is clear that these core stem cell factors are essential for maintaining the self-renewal and pluripotency of ESCs, CSCs, and adult stem cells [11–14]. In squamous-cell carcinoma, SOX2 is required for the stemness of CSCs [14]. Transcription factor TGLI1 activates CSCs in the tumor microenvironment to mediate breast cancer brain metastasis [15]. In melanoma and breast cancer stem cells, transcription factor YB-1 plays an important role in maintaining the stemness of CSCs [16]. These findings show that transcription factors play essential roles in the stemness of CSCs. Usually, transcription factors bind to specific promoters to regulate the expressions of protein-encoding genes. Interestingly, our previous study reveals that transcription factor NME2 (NME/NM23 nucleoside diphosphate kinase 2) is a master suppressor for apoptosis of gastric cancer cells via regulating the expressions of miRNA and protein-encoding genes [17]. At present, however, the underlying mechanism of transcription factors in CSCs is still poorly understood. In this context, transcription factor NME2 merits to be characterized in CSCs.

To address this issue, the mechanism of NME2 in the maintenance of stemness of gastric cancer stem cells was explored in the present study. The results showed that NME2 was upregulated in gastric cancer stem-like cells. The in vitro and in vivo data revealed that NME2 was required for the maintenance of gastric cancer stem-like cell stemness by promoting the expressions of anti-apoptosis genes.

## Materials and methods

### Cell viability analysis

CellTiter 96 Aqueous One Solution Assay (Promega, USA) was used to detect cell viability. The suspended cells (100 μL) were mixed with 20 μL CellTiter 96 Aqueous One Solution Reagent and then incubated for about 1 h. Finally, the OD value of cells was detected at 490 nm.

### Cell cycle analysis

Cells were placed at −20 °C in 70% ethanol overnight, and then centrifuged and resuspended with 0.5 mL PBS. Subsequently, 20 μg/mL DNase-free RNase A was added to the cells, followed by culture at 37 °C for 30 min. After the addition of 50 μg/mL propidium iodide, the fluorescence intensity of cells was detected by flow cytometry.

### Western blot analysis

Proteins were separated by gel electrophoresis (15% sulfate polyacrylamide gel) and then the separated proteins were transferred to polyvinylidene difluoride membrane (Millipore, USA). Five percent skim milk was dissolved in triethanolamine-buffered saline to block the membrane. After incubation with primary antibody (Abcam, Shanghai) over night at 4 °C, the membrane was incubated with the secondary antibody (Roche, Switzerland) for 2 h at room temperature. The signals were detected with BCIP/NBT substrate (Sangon Biotech, Shanghai, China).

### Tumorigenicity in immunodeficient mice

Cancer stem-like cells and cancer non-stem-like cells (5 × 10^3^ cells/mL) were resuspended in physiological saline and then mixed with Matrigel (Becton, Dickinson and Company, USA), followed by subcutaneous injection of 100 mL of cell suspension into five female non-obese diabetes/severe combined immunodeficiency (NOD/SCID) mice. Five to six weeks later, the mice were sacrificed and the solid tumors were taken out. Experiments on mice were conducted in accordance with the protocols approved by The China Institutional Animal Care and Use Committee (IACUC).

### Statistical analysis

All data in this study were mean ± SD (standard deviation). The significance of difference was analyzed by *t*-test and one-way ANOVA (analysis of variance).

## Results

### Sorting of gastric cancer stem-like cells

**Fig. 1:**
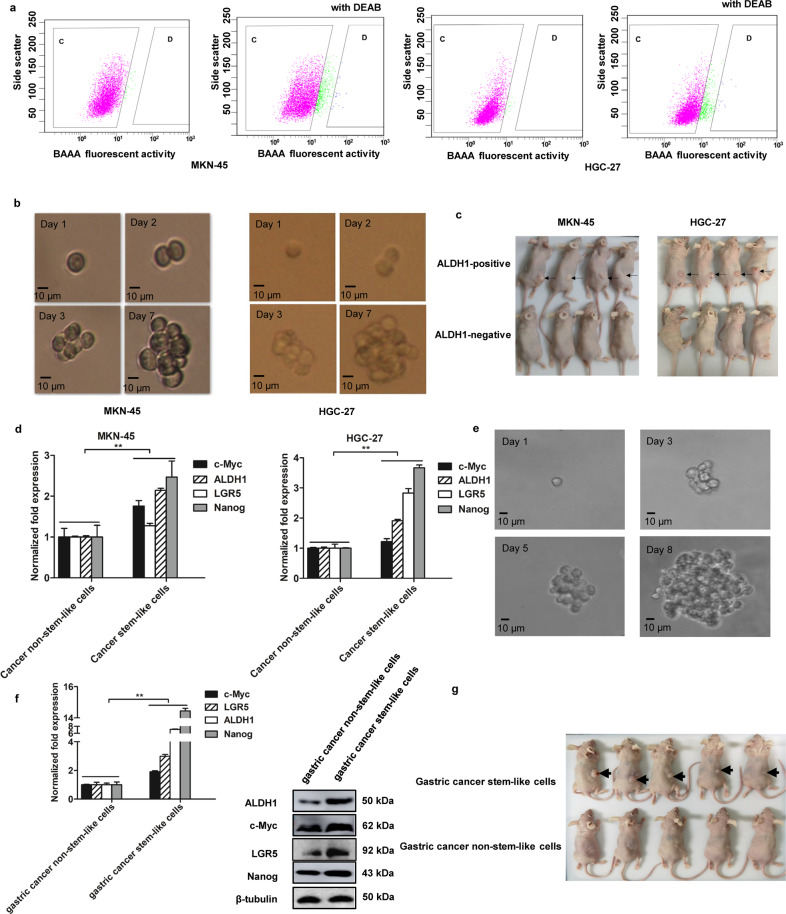
Sorting of gastric cancer stem-like cells. **a** Sorting of gastric cancer stem-like cells. The fluorescence activated cell sorting was performed based on the detection of ALDH1 activity using the ALDH1 fluorescent substrate BODIPY-aminoacate (BAAA). As a control, the activity of ALDH1 was inhibited by DEAB. The ALDH1-positive cells were potential gastric cancer stem-like cells (D region) and ALDH1-negative cells were cancer non-stem-like cells (C region). **b** Tumorsphere formation assay. The ALDH1-positive cells were subjected to tumorsphere formation assay. The sphere formation was examined with a light microscope. Scale bar, 100 μm. **c** Tumorigenicity of cancer stem-like cells in nude mice. Mice were subcutaneously injected with ALDH1-positive or ALDH1-negative cells. Forty days later, the tumors were examined. Arrows indicate the tumors. **d** Differential expressions of stemness-associated genes in gastric cancer stem-like cells and non-stem-like cells. Quantitative real-time PCR was conducted to detect the mRNA levels (^**^p < 0.01). **e** Tumorsphere formation of gastric cancer stem-like cells from the solid tumors of patients with gastric cancer. Scale bar, 10 μm. **f** The expression levels of stemness genes in gastric cancer stem-like cells isolated from the solid tumors of gastric cancer patients. The statistical significance of difference between treatments was indicated with asterisks (^**^p < 0.01). **g** Tumorigenicity of the potential gastric cancer stem-like cells in mice. Nude mice were subcutaneously injected with the potential gastric cancer stem-like cells or gastric cancer non-stem-like cells. Forty days later, the tumors were examined. Arrows indicate the tumors.

To obtain gastric cancer stem-like cells, the gastric cancer stem-like cells and non-stem-like cells were sorted from MKN-45 and HGC-27 cells using ALDH1, which was a marker of cancer stem-like cells. The ALDH1-positive cells were the potential gastric cancer stem-like cells (Fig. 1a, D region), whereas the ALDH1-negative cells were gastric cancer non-stem-like cells (Fig. 1a, C region). The ALDH1-positive cells were grown under serum-free conditions with recombinant growth factors. Cultured CSCs can form spheres in vitro and form tumor in vivo, which are very similar to endogenous CSCs isolated from human tumor tissues [18]. Thus, the isolated ALDH1-positive cells from gastric cell lines were cultured in a serum-free medium. About 7 days later, a single ALDH1-positive tumor cell formed a sphere (Fig. 1b). Then, the sphere was scattered in DMEM/ F-12 medium, followed by tumorsphere formation assay with a single cell for three times. The tumorsphere formation assays generated the same results as shown in Fig. 1b, suggesting that these ALDH1-positive tumor cells might be cancer stem-like cells.

To assess the ability of tumor formation of ALDH1-positive cells in vivo, 500 ALDH1-positive cells from tumorspheres were injected into NOD/SCID mice. The data demonstrated that solid tumors formed in the mice injected with ALDH1-positive cells, while no tumor was observed in the mice treated with the ALDH1-negative cells (Fig. 1c). These results showed that the ALDH1-positive cells had key features consistent with stem cells, indicating that the ALDH1-positive cells were gastric cancer stem-like cells.

To further confirm the gastric cancer stem-like cells, the expressions of c-Myc, Nanog, ALDH1, and LGR5, the markers of gastric cancer stem cells [16, 19, 20], were examined in gastric cancer stem-like cells. The results of quantitative real-time PCR indicated that the four stemness-associated genes were significantly upregulated in the gastric cancer stem-like cells compared with those of cancer non-stem-like cells (Fig. 1d). These data demonstrated that gastric cancer stem-like cells were obtained from gastric cancer cells (MKN-45 and HGC-27).

To obtain gastric cancer stem cells from solid tumors, the gastric cancer stem cells were sorted from the solid tumors of gastric cancer patients. The results revealed that a single tumor cell of the sorted cells could form a sphere (Fig. 1e). The stemness genes (c-Myc, LGR5, ALDH1, and Nanog) were significantly upregulated in the potential gastric cancer stem cells compared with gastric cancer non-stem-like cells (Fig. 1f). At the same time, the tumorigenicity assays of the sorted cells in mice revealed that the potential gastric cancer stem cells formed solid tumors in mice, while no tumor was observed in mice injected with gastric cancer non-stem cells (Fig. 1g). These findings indicated that gastric cancer stem-like cells were obtained from the solid tumors of gastric cancer patients.

### Upregulation of NME2 in gastric cancer stem-like cells

**Fig. 2:**
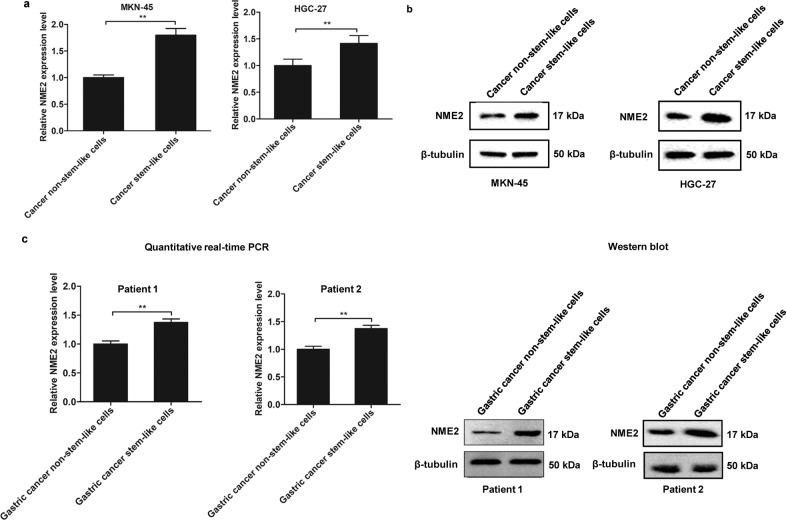
Upregulation of NME2 in gastric cancer stem-like cells. **a** Differential expression of NME2 in gastric cancer stem-like cells and cancer non-stem-like cells. Quantitative real-time PCR was conducted to detect the NME2 mRNA level. **b** Western blot analysis of NME2 protein in gastric cancer stem-like cells and cancer non-stem-like cells. β-Tubulin was used as a control. **c** The expression of NME2 in gastric cancer stem-like cells or cancer non-stem-like cells sorted from the solid tumor of two gastric cancer patients. The mRNA level and the protein level of NME2 were examined with quantitative real-time PCR (left) and western blot (right), respectively.

To evaluate the role of NME2 in the progression of gastric cancer, the expression level of NME2 in gastric cancer stem-like cells and non-stem-like cells, sorted from MKN-45 and HGC-27 cell lines, was characterized. Quantitative real-time PCR data indicated that NME2 was significantly upregulated in gastric cancer stem-like cells compared with cancer non-stem-like cells (Fig. 2a). Western blots generated the similar results (Fig. 2b), suggesting that NME2 played an important role in gastric cancer stem-like cells.

To characterize the NME2 expression in gastric cancer stem-like cells sorted from the solid tumors of two gastric cancer patients, the mRNA and protein of NME2 was examined. It was found that NME2 was significantly upregulated in gastric cancer stem-like cells compared with gastric cancer non-stem-like cells (Fig. 2c).

### Influence of NME2 on the stemness of gastric cancer stem-like cells

**Fig. 3:**
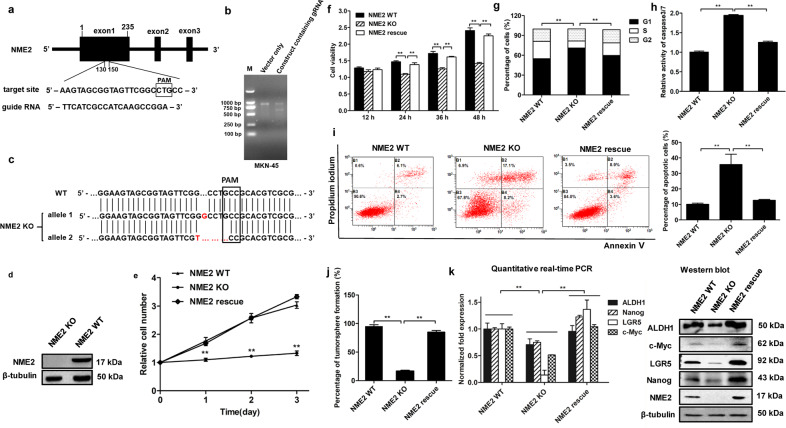
Influence of NME2 on the stemness of gastric cancer stem-like cells. **a** Schematic representation of the guide RNA targeting NME2 gene. The sequence of PAM (protospacer adjacent motif) was boxed. **b** T7 endonuclease 1(T7E1) assay. Gastric cancer stem-like cells (MKN-45) were transfected with pHBCas9/gRNA-Pure plasmid containing NME2 gRNA. As a control, vector only (without NME2 gRNA) was included in the transfection. The DNA containing target site of NME2 gRNA was amplified by PCR using the genomic DNA extracted from the transfected cells. The PCR product was digested with T7E1, followed by agarose gel electrophoresis. M, DNA marker. **c** Sequencing analysis of NME2 in wild-type (WT) and NME2-knocked out (KO) gastric cancer stem-like cells. The sequences of two alleles of NME2 mutant were indicated. PAM was boxed. **d** Western blot analysis of NME2 in NME2-knockout (NME2 KO) gastric cancer stem-like cells. β-Tubulin was used as a control. **e** Impact of NME2 knockout and rescue on the cell number of gastric cancer stem-like cells. The cells transfected with vector alone or pcDNA-NME2 plasmid were cultured and counted at 1st, 2nd, and 3rd days (^**^p < 0.01). **f** Influence of NME2 knockout and rescue on the cell viability of gastric cancer stem-like cells. The cells transfected with vector alone or pcDNA-NME2 plasmid were cultured for different times, followed by the examination of cell viability (^**^p < 0.01). **g** Impact of NME2 knockout and rescue on cell cycle of gastric cancer stem-like cells. The gastric cancer stem-like cells containing WT, KO, or rescue NME2 were cultured for 48 h and then subjected to cell cycle examination with flow cytometry (^**^p < 0.01). **h** Effects of NME2 knockout or rescue on apoptosis of gastric cancer stem-like cells. The cells with different treatments were cultured for 48 h. Subsequently, the caspase 3/7 activity of the cells was examined (^**^p < 0.01). **i** Detection of apoptosis using Annexin V (^**^p < 0.01). **j** Tumorsphere formation assay of the NME2 -knocked out or -rescued gastric cancer stem-like cells. At day 8 after the inoculation of a single cell, the percentage of tumorsphere formation was calculated (^**^p < 0.01). **k** Effects of NME2 knockout and rescue on the expressions of stemness genes in gastric cancer stem-like cells. The cells with different treatments were cultured for 48 h, followed by quantitative real-time PCR and western blot (^**^p < 0.01).

To explore the impact of NME2 on cancer stem-like cells, the NME2 gene was knocked out in gastric cancer stem-like cells (MKN-45) using CRISPR/Cas9 system. A guide RNA (gRNA) was designed, which was located at 130–150 bp of NME2 exon1 (Fig. 3a). The data revealed that the DNA, amplified from the gRNA-transfected gastric cancer stem-like cells, was cleaved into two bands by T7E1 enzyme compared with the control (Fig. 3b), showing that the NME2 gRNA was introduced into the genome of gastric cancer stem-like cells. The sequencing results showed that the two alleles of NME2 gene were knocked out in gastric cancer stem-like cells (Fig. 3c). Western blot data demonstrated that the NME2 protein could not be detected in NME2-knockout gastric cancer stem-like cells (Fig. 3d). These data revealed that the NME2-knockout gastric cancer stem-like cells were generated.

The data of cell counting and MTS assays presented that the NME2 knockout (KO) resulted in a significant decrease of the number and cell viability of gastric cancer stem-like cells, while the cell number and viability of the cells with NME2 rescue treatment were comparable to those of NME2 wild-type (WT) cells (Fig. 3e, f). These results showed that NME2 could promote the proliferation of gastric cancer stem-like cells.

To reveal the mechanism of cell proliferation promotion of gastric cancer stem-like cells by NME2, the cell cycle was analyzed with flow cytometry. The data revealed that the percentage of NME2 KO cells in the G1 phase was significantly increased compared with that of wild-type cells (Fig. 3g). The percentage of NME2 rescue cells in the G1 phase was comparable to that of wild-type gastric stem-like cells (Fig. 3g). These results demonstrated that the loss of NME2 resulted in cell cycle arrest in G1 phase.

The detection of caspase 3/7 activity showed that the NME2 KO significantly increased the caspase 3/7 activity of NME2 KO compared with NME2 WT, while the NME2 rescue generated the similar results to NME2 WT (Fig. 3h). The Annexin V assays yielded the similar results (Fig. 3i). These data indicated that the NME2 KO could promote apoptosis of gastric cancer stem-like cells.

Tumorsphere formation assay was performed to examine the effect of NME2 on the tumorsphere capacity. It was found that, compared with NME2 WT cells, the percentage of tumorsphere formation of NME2 KO cells have dropped to 16.67%, while the percentage of NME2 rescue cells was relatively flat (Fig. 3j). Then to evaluate the role of NME2 in regulating the stemness of gastric cancer stem-like cells, the expression levels of cancer stemness genes were examined. Quantitative real-time PCR and western blot results showed that the expressions of cancer stemness genes were significantly decreased in NME2 KO compared with NME2 WT, while NME2 rescue significantly increased the expressions of cancer stemness genes (Fig. 3k), indicating that NME2 had a vital effect on the stemness of gastric stem-like cells.

### Effects of NME2 on gastric cancer stem-like cells from solid tumors of patients

**Fig. 4:**
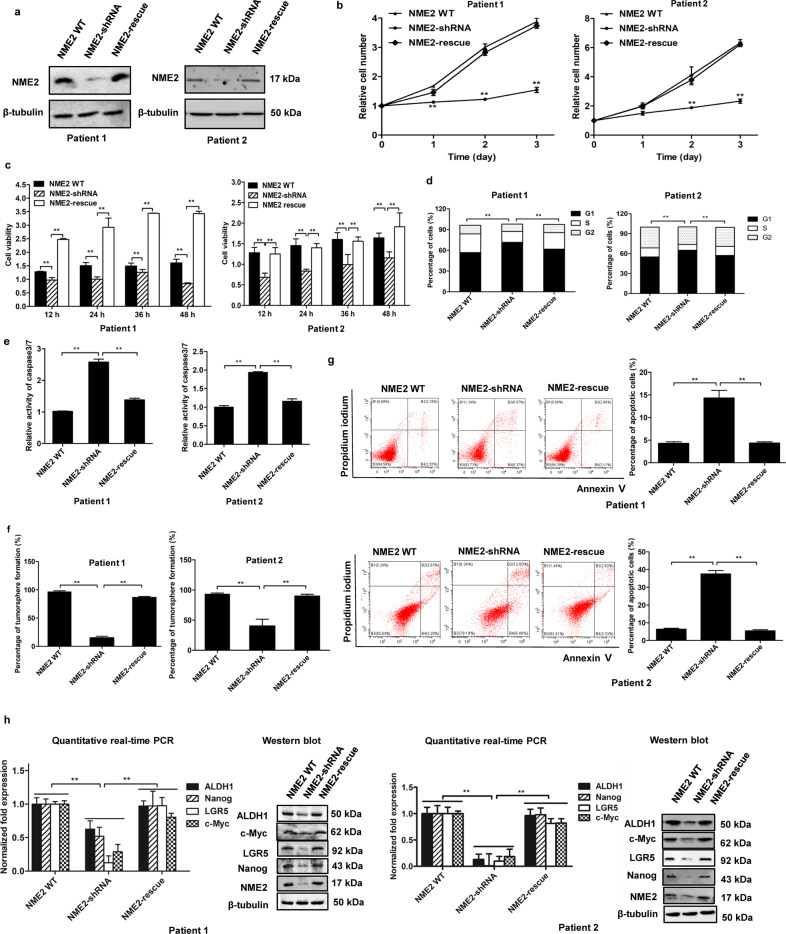
Effects of NME2 on gastric cancer stem-like cells from solid tumors of patients. **a** The expression level of NME2 protein in NME2-silenced or -rescued in gastric cancer stem-like cells sorted from solid tumors of two gastric cancer patients. The protein was examined by western blot. β-Tubulin was used as a control. **b** Impact of NME2 knockdown and rescue on the cell number of gastric cancer stem-like cells. The gastric cancer stem-like cells sorted from solid tumors were transfected with NME2-shRNA to silence the NME2 expression. To rescue the NME2 expression, the NME2-silenced cells were transfected with the plasmid expressing NME2. At different days after transfection, the cell number was counted (^**^p < 0.01). **c** Influence of NME2 knockdown and rescue on the cell viability of gastric cancer stem-like cells. The NME2-silenced cells were transfected with the plasmid expressing NME2, and the cell viability was examined at different times after transfection (^**^p < 0.01). **d** Role of NME2 in cell cycle of gastric cancer stem-like cells sorted from solid tumors of two gastric cancer patients. The NME2-knock-down cells were transfected with the plasmid expressing NME2. At 48 h after transfection, the cells were subjected to the examination of cell cycle (^**^p < 0.01). **e** Impact of NME2 knockdown and rescue on the caspase 3/7 activity of gastric cancer stem-like cells. The NME2-knocked-down cells were transfected with the plasmid expressing NME2. At 48 h after transfection, the caspase 3/7 activity of cells was examined (^**^p < 0.01). **f** Flow cytometry detection of the impact of NME2 knockdown and rescue on apoptosis of gastric cancer stem-like cells sorted from solid tumors. The cells transfected with the plasmid expressing NME2 were cultured for 48 h, followed by the examination of apoptosis using Annexin V (^**^p < 0.01). **g** Tumorsphere formation assay of the NME2-silenced or -rescued gastric cancer stem-like cells sorted from solid tumors. At day 8 after the inoculation of a single cell, the percentage of tumorsphere formation was calculated (^**^p < 0.01). **h** Influence of NME2 knockdown and rescue on the expressions of stemness genes in gastric cancer stem-like cells from solid tumors. At 48 h after NME2 rescue, the expressions of stemness genes were examined with quantitative real-time PCR or western blot (^**^p < 0.01).

To reveal the role of NME2 in gastric cancer stem-like cells from solid tumors, the NME2 expression was knocked down or rescued in gastric cancer stem-like cells sorted from solid tumors of two gastric cancer patients (Fig. 4a). The results of cell counting and MTS assays showed that NME2 played an important role in the proliferation and growth of gastric cancer stem-like cells sorted from solid tumors (Fig. 4b, c).

To explore the influence of NME2 on the cell cycle process of gastric cancer stem-like cells sorted from solid tumors, the cell cycle of NME2-silenced or -rescued cells was examined. The results demonstrated that the NME2 knockdown led to cell cycle arrest in G1 phase, while the cell cycle of NME2-rescued cells was similar to that of the wild-type cells (Fig. 4d), showing that the NME2 silencing suppressed the proliferation of gastric cancer stem-like cells.

To evaluate whether the suppression of cell cycle by NME2 silencing led to apoptosis of gastric cancer stem-like cells, the activity of caspase 3/7 of gastric cancer stem-like cells with different treatments was examined. The results demonstrated that the NME2 knockdown significantly increased the caspase 3/7 activity of gastric cancer stem-like cells compared with the control, while the caspase 3/7 activity of the NME2-rescue cells was comparable to that of the control (Fig. 4e). The Annexin V assays essentially generated the similar results (Fig. 4f). These data showed that the suppression of cell proliferation by NME2 silencing promoted apoptosis of gastric cancer stem-like cells.

To explore the impact of NME2 on tumorigenesis of gastric cancer stem-like cells sorted from solid tumors of two gastric cancer patients, the tumorsphere formation capacity of the NME2-silenced or -rescued cells was examined. The results showed that the percentage of tumorsphere formation of NME2-silenced cells was significantly decreased compared with the control, while the rescue of NME2 in NME2-silenced cells significantly increased the tumorsphere formation capacity (Fig. 4g). Meanwhile, the expression levels of stemness genes were significantly decreased in the NME2-silenced cells and the expression profiles of stemness genes were comparable to those of the control (NME2 WT) (Fig. 4h). These data revealed that that NME2 played an important role in the maintenance of stemness of gastric cancer stem-like cells from solid tumors.

Taken together, these findings demonstrated that NME2 was critical for the maintenance of stemness of gastric cancer stem-like cells from solid tumors of gastric cancer patients by suppressing apoptosis of CSCs.

### Role of NME2 in tumorigenesis of gastric cancer stem-like cells in vivo

**Fig. 5:**
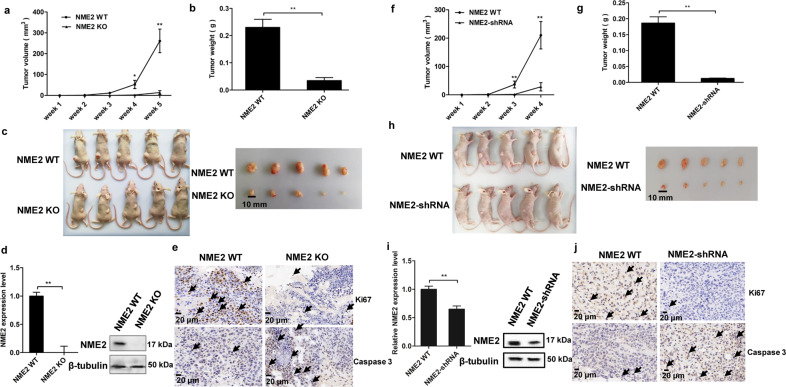
Role of NME2 in tumorigenesis of gastric cancer stem-like cells in vivo. **a** Effects of NME2 knockout on tumor growth in nude mice. NME2-knockout (NME2 KO) and wild-type (NME2 WT) cancer stem-like cells (MKN-45) were injected into nude mice, respectively. The tumor volume of mice was examined every week after cell inoculation. Five weeks later, the mice were sacrificed. The mean of five mice was indicated (^*^p < 0.05; ^**^p < 0.01). **b** Evaluation of solid tumor size derived from mice. At 5 weeks after cell inoculation, the mice were sacrificed and the tumor sizes were examined. **c** Solid tumor weight of the mice with different treatments (^**^p < 0.01). **d** Examination of NME2 in the soli**d** tumors of mice with western blot and quantitative real-time PCR (^**^p < 0.01). In western blot, β-tubulin was used as a control. **e** Immunohistochemical analysis of the expressions of Ki67 and caspase 3 in the solid tumors of the mice injected with NME2-knockout cells (NME2 KO) or with wild-type NME2 cells (NME2 WT). Brown indicated the target proteins and blue represented nuclei stained by hematoxylin. The arrows showed the target proteins. Scale bar, 20 μm. **f** Impact of NME2 knockdown on tumor growth in nude mice. The gastric cancer stem-like cells sorted from solid tumors of patients were used in the assays. The tumor volume was measured every week. The mean of five mice was showed (^**^p < 0.01). **g** Solid tumor size. At 4 weeks after cell inoculation, the mice were sacrificed. **h** Influence of NME2 knockdown on tumor weight. The difference between treatments was indicated with asterisks (^**^p < 0.01). **i** Expression level ofNME2 in the solid tumors of mice. β-Tubulin was used as a control (^**^p < 0.01). **j** Immunohistochemical analysis of the expression of Ki67 and caspase 3 in solid tumors of the mice injected with NME2-silenced cells (NME2-shRNA) or with wild-type NME2 cells (NME2 WT). Brown represented the target proteins and blue represented nuclei stained by hematoxylin. The target proteins were also indicated with arrows. Scale bar, 20 μm.

To investigate the impact of NME2 on tumorigenesis of gastric cancer stem-like cells in vivo, the NME2-knockout (KO) and wild-type cancer stem-like cells sorted from MKN-45 cells were injected into nude mice, respectively. The results showed that the tumor growth of the mice injected with NME2-knockout cells (NME2 KO) was significantly suppressed compared with that of the mice injected with wild-type NME2 cells (NME2 WT) (Fig. 5a). The sizes and weights of solid tumors of the mice injected with NME2 KO were much smaller and lower than those of the mice injected with NME2 WT (Fig. 5b, c). At the same time, western blot and quantitative real-time PCR revealed that NME2 was not detected in the solid tumors of the mice injected with NME2 KO (Fig. 5d). These data showed that NME2 played a positive role in tumorigenesis of gastric cancer stem-like cells in vivo. The immunohistochemical results showed that caspase 3 was significantly upregulated in the solid tumors of the mice injected with NME2 KO, while Ki67 was not detected in the NME2 KO treatment (Fig. 5e), indicating that NME2 was required for anti-apoptosis of gastric cancer stem-like cells.

To evaluate the effects of NME2 on tumorigenesis of gastric cancer stem-like cells sorted from solid tumors of patients in vivo, the NME2-silenced cancer stem-like cells were injected into five nude mice. The results revealed that the tumor growth in the mice treated with NME2-shRNA was significantly suppressed compared with the control (Fig. 5f). The examination of tumor size and tumor weight essentially generated the similar results (Figs. 5g, h). In the tumors of mice, the NME2 expression level was significantly decreased (Fig. 5i). These findings indicated that the NME2 silencing led to the suppression of tumorigenesis of gastric cancer stem-like cells sorted from solid tumors of patients. Based on the immunohistochemical analysis, it was revealed NME2 played a positive role in anti-apoptosis of gastric cancer stem-like cells (Fig. 5j).

These findings demonstrated that the NME2 silencing could trigger apoptosis of gastric cancer stem-like cells, thus suppressing tumorigenesis in vivo.

### Mechanism of NME2-mediated apoptosis in gastric cancer stem-like cells from solid tumors of patients

**Fig. 6:**
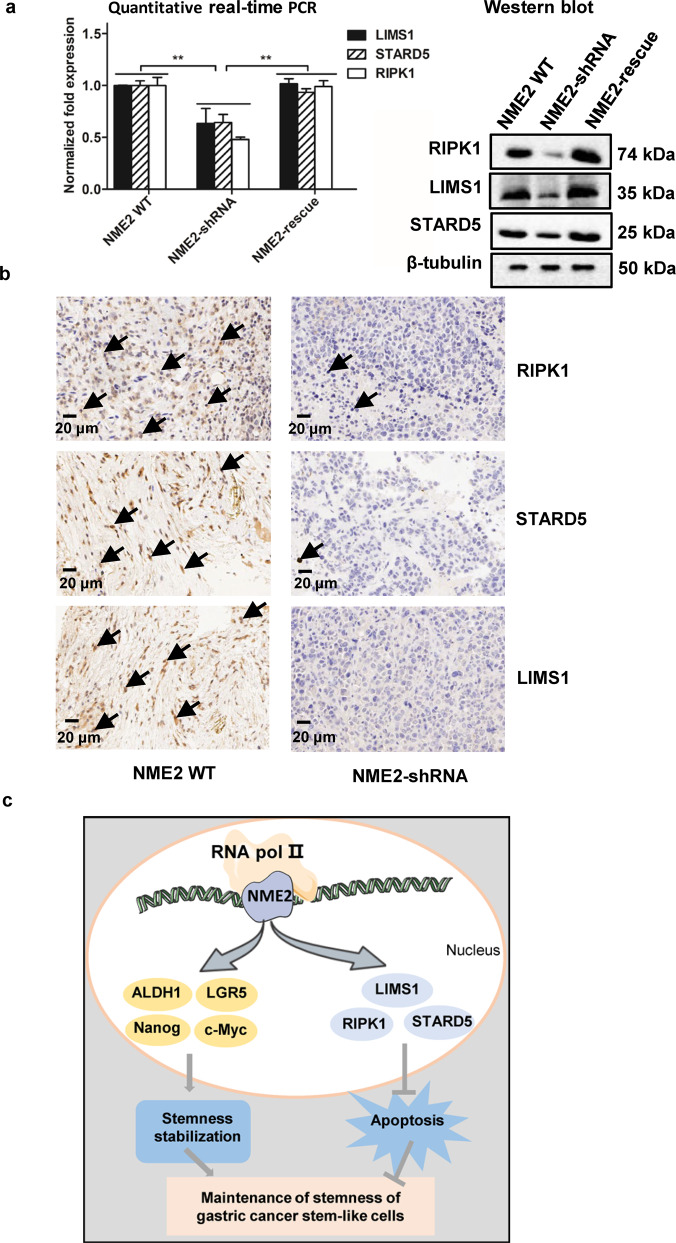
Mechanism of NME2-mediated apoptosis in gastric cancer stem-like cells from solid tumors of patients. **a** Effects of NME2 silencing and rescue on the expressions of anti-apoptosis genes in gastric cancer stem-like cells sorted from the solid tumors of patients. The gastric cancer stem-like cells were transfected with NME2-shRNA to silence the NME2 expression. To rescue the NME2 expression, the NME2-silenced cells were transfected with the plasmid expressing NME2. At 48 h after NME2 rescue, the expression levels of RIPK1, STARD5and LIMS1 were examined with western blot and quantitative real-time PCR (^**^p < 0.01). In western blot, β-tubulin was used as a control. **b** Immunohistochemical analysis of the expressions of anti-apoptosis genes (LIMS1, RIPK1, and STARD5) in the solid tumors of mice. The NME2-knocked-down gastric cancer stem-like cells from the solid tumors of patients were injected into nude mice. Four weeks later, the solid tumors of mice were subjected to immunohistochemical analysis. Brown indicated the proteins labeled and blue represented nuclei stained by hematoxylin. Scale bar, 20 μm. **c** Model for the mechanism of the NME2-mediated maintenance of stemness of gastric cancer stem-like cells.

To reveal the mechanism of NME2 in gastric cancer stem-like cells, the genes regulated by NME2 were analyzed. As reported, NME2, a transcription factor, is responsible for the transcription of RIPK1, STARD5, and LIMS1 [17], while RIPK1, STARD5, and LIMS1 play positive roles in anti-apoptosis [21–23]. Thus, the influence of NME2 on the expressions of these genes in gastric cancer stem-like cells sorted from the solid tumors of gastric cancer patients was characterized. The results of western blot and quantitative real-time PCR showed that the NME2 knockdown significantly downregulated the expression levels of RIPK1, STARD5, and LIMS1 in gastric cancer stem-like cells from human solid tumors (Fig. 6a). The NME2 rescue could recover the expressions of RIPK1, STARD5, and LIMS1 (Fig. 6a). At the same time, the immunohistochemical analysis demonstrated that the NME2 silencing significantly downregulated the expressions of LIMS1, RIPK1, and STARD5 in the solid tumors of mice (Fig. 6b). These data indicated that NME2 could promote the expression of anti-apoptosis genes in cancer stem-like cells, thus suppressing apoptosis of gastric cancer stem-like cells.

The above data presented that NME2 played an important role in the gastric cancer stem cell stemness (Figs. 3k and 5h). Collectively, NME2 was critical for the maintenance of stemness of gastric cancer stem-like cells via promoting the expression of stemness-associated transcriptional factors (c-Myc, LGR5, ALDH1, and Nanog) and anti-apoptosis genes (RIPK1, STARD5, and LIMS1) (Fig. 6c).

## Discussion

The abnormal expression of genes can lead to the biogenesis of CSCs, thus contributing to tumorigenesis and development [24]. Transcription factors, irreplaceable proteins in the regulation of gene expression, play important roles in tumorigenesis by binding to *cis*-acting elements of genes to respond to external stimuli or environmental stresses [25, 26]. Thus, the roles of transcription factors in the biogenesis of CSCs attract more and more attention. In the present study, the results indicated that the transcription factor NME2 played a positive role in the maintenance of the stemness of gastric cancer stem-like cells sorted from gastric cancer patients. NME2 promoted the proliferation and suppressed apoptosis of gastric cancer stem-like cells, thus being involved in tumorigenesis of gastric cancer. Therefore, our study presented a novel transcription factor responsible for the maintenance of the stemness of gastric cancer stem-like cells. It is reported that NME2 can inhibit the transcriptional activity of human telomerase reverse transcriptase in a G-quadruplex-dependent manner in two tumor cell lines including HT-1080 fibrosarcoma cells and HCT116 colon cancer cells [27]. In A549 cell line, the NME2 downregulation causes transcriptional de-repression of vinculin, thus promoting lung cancer metastasis [28]. In gastric cancer cell lines (HGC-27 and MKN-45), NME2 functions as a master suppressor for apoptosis of gastric cancer by interacting with RNA polymerase II and RNA polymerase II-associated protein 2 (RPAP2) [17]. These investigations are conducted in tumor cell lines, while our findings are obtained using gastric cancer stem-like cells from patients. In this context, our findings contributed novel insights into the mechanisms of tumorigenesis of gastric cancer stem cells.

Due to the importance of apoptosis, tumorigenesis is usually accompanied by the inhibition of apoptosis, which is usually achieved by abnormal expression of a variety of tumor suppressor genes, such as Tp53 gene [29, 30]. At present, however, the regulatory mechanism of apoptosis in CSCs has not been extensively explored. In this study, the findings revealed that the transcription factor NME2 could suppress apoptosis of gastric cancer stem-like cells via promoting the expression of anti-apoptosis genes (RIPK1, STARD5, and LIMS1), indicating that NME2 was an anti-apoptosis transcription factor in gastric cancer stem cells. Up to date, some anti-apoptosis transcription factors, including OCT4, NFAT (nuclear factor of activated T cells), and STAT3 (signal transducer and activator of transcription 3) [31–33], are found to promote tumorigenesis. In the CSCs sorted from human ovarian cancer SKOV3 and A2780 cell lines, OCT4 can promote tumorigenesis by increasing the phosphorylation levels of JAK1 and STAT6 proteins of JAK/STAT signaling pathway [31]. NFAT is a repressor of cell death and a positive regulator of cell proliferation in NIH3T3 cell line [32]. STAT3 can be activated by the phosphorylation of its tyrosine and serine residues after receiving signals from upstream regulators, and the activation of STAT3 inhibits apoptosis of different classical Hodgkin lymphoma cell lines [33]. As reported, NME2 can suppress the metastasis and invasion of cancer cells [28, 34, 35]. The inhibition of tumor metastasis and invasion may result from apoptosis of cancer cells. In this context, our study revealed a novel anti-apoptosis transcription factor, which functioned in the gastric cancer stem-like cells sorted from the solid tumors of gastric cancer patients. NME2 could be a target for the diagnosis and therapy of gastric cancers.

## Conclusions

Transcription factor NME2 was vital for the maintenance of gastric cancer stem-like cells via regulating anti-apoptosis pathway and promoting stemness-associated genes’ expressions.

## Supplementary information

Supplementary information 1.

## Data Availability

The datasets used during the current study are available from the corresponding author on reasonable request.
