# Supplementary material for: Requirement of transcription factor NME2 for the maintenance of the stemness of gastric cancer stem-like cells
Source: Cell Death Dis. 2021 Oct 9;12(10):924. doi: 10.1038/s41419-021-04234-1 (PMC8502175; doi:10.1038/s41419-021-04234-1)
Supplement: Supplementary file 1 — Supplementary information 1. [file 41419_2021_4234_MOESM1_ESM.docx]

**Materials and methods**

**Sorting and culture of cancer stem-like cells**

Sorting of gastric cancer stem-like cells was carried out under the guidance of ALDEFLUOR kit (Cyagen Biosciences Inc., USA) by detecting ALDH1 (aldehyde dehydrogenase 1). MKN-45 and HGC-27 cells were cultured in ALDEFLUOR assay buffer with 1 mmol/L BAAA (ALDH1 fluorescent substrate BODIPY amino acetate) at 37°C for 45 min. At the same time, the same amounts of cells was treated with the specific ALDH1 inhibitor DEAB (diethylaminobenzaldehyde, 50 mmol/L) as a negative control. After that, the cells were resuspended with 500 μL ALDEFLUOR assay buffer and subject to FACS (fluorescence-activated cell sorting). FACS was performed at 575 nm excitation.

To sort cancer stem-like cells from the solid tumors of gastric cancer patients, the cancerous tissues were immediately washed using the mild-washing solution [DMEM (Dulbecco’s modified Eagle medium) added with 10% FBS (fetal bovine serum) and 100 μg/mL penicillin-streptomycin] (Gibco, U.S.A). Subsequently the tissues were cut into pieces, and rinsed three times using phosphate-buffered saline (PBS), and it’s better to add 100 μg/mL penicillin-streptomycin to PBS. After low-speed centrifugation, the pellet was resuspended in the mild-washing solution containing 0.5% of collagenase, followed by incubation for 1-4 h in 37℃ with 5% CO_2_. When the cells were fully digested, the cells were rinsed using the mild-washing solution for 2-3 times. The cells were then cultured in DMEM medium with 1% minimum essential medium (MEM) (Gibco, U.S.A), 20% FBS, 100 μg/mL penicillin-streptomycin, 1% glutamic acid (Gibco, U.S.A) and 1% alanine (Gibco, U.S.A) at 37 ℃ with 5% CO_2_. Two weeks later, the cells were seeded into an ultra-low attachment six-well plateat a density of 1×10^3^ cells/well and cultured in specific stem cell culture medium made from serum-free DMEM/F-12 medium added with 2% B27 (Invitrogen, USA), 5 μg/mL insulin (MedChem Express, U.S.A), 20 ng/mL epidermal growth factor (MedChem Express), 100 μg/mL penicillin-streptomycin and 10 ng/mL basic fibroblast growth factor (MedChem Express) in 37℃ incubator with 5% CO_2_. One week later, a single cell was picked out from a tumorshpere and seeded into a new ultra-low attachment plate. This procedure was repeated for 3 times to obtain the cancer stem-like cells. The solid tumors were provided by The First Affiliated Hospital, Zhejiang University School of Medicine.

**Establishment of NME2 knockout mutant of gastric cancer stem-like cells**

NME2-specifc guide RNA (gRNA) (5’-TTCATCGCCATCAAGCCGGA-3’) was designed and cloned into pHBCas9/gRNA-Pure vector (Hanheng Biotechnology, Shanghai, China). Gastric cancer stem-like cells were then transfected with these recombinant plasmids by Lipofectamine 2000 (Invitrogen, U.S.A) and screened by 0.8 μg/mL puromycin. To estimate the gRNA activity, the genomic NME2 of transfected cells was amplified using NME2-specific primers (5’-ATGGTGGTCGCACCAGCTCTCTGCTC-3’ and 5’-CGAGTCACGCTACCAG CGCTTCACC-3’). The amplified DNA was digested using T7E1 (T7 endonuclease 1, New England Biolabs, U.S.A) for 15 min, and analyzed by agarose gel electrophoresis. After screening, single colonies were selected and passaged respectively. After the DNA sequencing and Western blot verification, the NME2 knockout mutant (MKN-45^NME2-/-^) was finally established.

**Tumorsphere formation assay**

Multiple single gastric cancer stem-like cells were inoculated into an ultra-low attachment 96-well culture plate (one cell per well) and cultured in DMEM/F-12 medium (Invitrogen, USA) supplemented with 20 ng/mL epidermal growth factor (Beyotime, China), 10 ng/mL basic fibroblast growth factor (Beyotime, China), 5μg/mL of insulin (Beyotime, China) and 2% of B27 (Sigma, USA). About 2 weeks later, spheroid colonies were examined using a light microscope. The forming spheres were scattered in DMEM/F-12 medium (Invitrogen). Subsequently, the tumorsphere formation assay was conducted with a single cell for three times.

**Quantitative real-time PCR**

RNA Isolation kit (Ambion, USA) was used to extract total RNAs and PrimeScript RT Reagent Kit (Vazyme, Nanjing, China) was employed to obtain cDNA. Hieff SYBR green Master Mix (YEASEN) was used for quantitative real-time PCR (qRT-PCR). The primers used were Nanog, 5’-TTCTTGACTGGGACCTTGTC-3’ and 5’-GCTTGCCTTGCTTTGAAGCA-3’; ALDH1, 5’-TTACCTGTCCTACTCACCGA-3’ and 5’-CTCCTTATCTCCTTCTTC TACCT-3’; GAPDH, 5’-GGTATCGTGGAAGGACTCATGAC-3’ and 5’-ATGCCAG TGAGCTTCCCGTTCAG-3’; LGR5, 5’-CAGGGAGTGGATTCTATTGTTATGG -3’ and 5’-AATCCCCGTCCAGGCTTTTAG -3’; OCT4, 5’-GAGCAAAACCCGGAG GAGT-3’ and 5’-TTCTCTTTCGGGCCTGCAC-3’; c-Myc, 5’- ACACCCGAGCAA GGACGCGA-3’ and 5’-C GCGGGAGGCTGCTGGTTTC-3’; NME2, 5’-CC ACCTCTTATTCATAGACCCA-3’ and 5’-AGATTCAAAGCCAGGCACCAT-3’; RIPK1, 5’-TCTGCTGGGAAGCGAATC-3’ and 5’-CATTTTCGTTTGAATAC TCTTT-3’; STARD5, 5’-CCTACGAGTGAAGTGGGATG-3’ and 5’-TGGCG TTGGAACTGATGG-3’; LIMS1, 5’-CATAATCGTGAGAAAGCCAGAG-3’ and 5’-GTGCATCGGCAGTTCGG-3. Relative fold change was calculated by 2^-(∆∆Ct)^ method.

**Detection of apoptosis**

To detect 3/7 activity, 1×10^4^/well cells were cultured to low-attachment 96-well plates for 2 days. Then equal volume of Caspase-Glo 3/7 reagent (Promega, U.S.A) was added to the cells. After incubation without light for 45 min, the luminescence was measured.

Apoptosis was also examined by Annexin V-FITC (fluorescein isothiocyanate) Apoptosis Kit (Becton, Dickinson and Company, U.S.A) using flow cytometry. Cells were stained with Alexa Fluor488 Annexin V and PI were according to the manufacturer’s protocol. The sample was analyzed with a flow cytometer at an excitation of 575 nm.

**Rescue of NME2 in NME2-knockout cells**

The NME2 gene was amplified with specific primers (5’-GAAAAGCTT GCCACCATGGCCAACCTGGAG-3’ and 5’-GAACTCGAGTTACTTATCGTCGTC ATCCTTGTAATCTTCATAGACCCAGTCATGAGCACAA-3’) and cloned into pcDNA3.1^+^ vector (Promega, U.S.A). The recombinant plasmids were transfected into NME2-knockout cancer stem-like cells to express NME2.

**NME2 knockdown and rescue in gastric cancer stem-like cells from solid tumors**

A small hairpin RNA (shRNA) targeting NME2 gene (5’-AAGAA CACCUGAAGCAGCACUACAU-3’) was cloned into the lentiviral vector LV2N (GenePharma, Shanghai, China). The recombinant vector was then transfected into HEK293T cells by Lipofectamine 2000 (Invitrogen, U.S.A) to package the virus. After transfecting for 48 h, the packaged virus was collected to infect gastric cancer stem-like cells. After purine screening, NME2 knockdown mutant was obtained.

To rescue the NME2 expression inNME2-silenced stem-like cells, the NME2 gene, which was mutated at position 283 from A to G to avoid the recognition by NME2-shRNA, was cloned into pcDNA3.1^+^ vector. Subsequently the recombinant plasmids were transfected into the NME2-knocked-down stem-like cells with Lipofectamine 2000 (Invitrogen, USA).
